# Intervening After Trauma: Child–Parent Psychotherapy Treatment Is Associated With Lower Pediatric Epigenetic Age Acceleration

**DOI:** 10.1177/09567976241260247

**Published:** 2024-08-14

**Authors:** Alexandra D. W. Sullivan, Sarah M. Merrill, Chaini Konwar, Michael Coccia, Luisa Rivera, Julia L. MacIsaac, Alicia F. Lieberman, Michael S. Kobor, Nicole R. Bush

**Affiliations:** 1 Department of Psychiatry and Behavioral Sciences, Weill Institute for Neurosciences, University of California, San Francisco; 2 BC Children’s Hospital Research Institute, Vancouver, British Columbia, Canada; 3 Department of Medical Genetics, University of British Columbia; 4 Centre for Molecular Medicine and Therapeutics, Faculty of Medicine, University of British Columbia; 5 Department of Psychiatry and Human Behavior, Warren Alpert Medical School of Brown University; 6 Neukom Institute for Computational Science, Dartmouth College; 7 Child and Brain Development Program, Canadian Institute for Advanced Research Institute, Toronto, Ontario, Canada; 8 Edwin S.H. Leong Centre for Healthy Aging, University of British Columbia; 9 Department of Pediatrics, Division of Developmental Medicine, University of California, San Francisco

**Keywords:** child–parent psychotherapy, PedBE, epigenetic age, intervention, childhood trauma

## Abstract

Early-life adversity increases the risk of health problems. Interventions supporting protective and responsive caregiving offer a promising approach to attenuating adversity-induced changes in stress-sensitive biomarkers. This study tested whether participation in an evidence-based dyadic psychosocial intervention, child–parent psychotherapy (CPP), was related to lower epigenetic age acceleration, a trauma-sensitive biomarker of accelerated biological aging that is associated with later health impairment, in a sample of children with trauma histories. Within this quasi-experimental, repeated-measures study, we examined epigenetic age acceleration at baseline and postintervention in a low-income sample of children receiving CPP treatment (*n* = 45; age range = 2–6 years; 76% Latino) compared with a weighted, propensity-matched community-comparison sample (*n* = 110; age range = 3–6 years; 40% Latino). Baseline epigenetic age acceleration was equivalent across groups. However, posttreatment, epigenetic age acceleration in the treatment group was lower than in the matched community sample. Findings highlight the potential for a dyadic psychosocial intervention to ameliorate accelerated biological aging in trauma-exposed children.

Early-life adversity is prospectively associated with serious and costly psychological- and physical-health problems (Grummitt et al., 2021; Ortiz et al., 2024; Yu et al., 2022). When responding to stress, children’s biology adapts to promote coping, regulation, and survival (for review, see Hays-Grudo et al., 2021; B. S. McEwen, 2007). However, stress that persists throughout *sensitive periods* in development—during which the brain is particularly susceptible to environmental experiences—may become *biologically embedded* (Bush, 2024; Shonkoff et al., 2009). In such cases, adversity may result in durable changes to stress-response systems, forecasting heightened adulthood disease risk.

Biological embedding is a process through which experiences of early-life adversity and trauma can “get under the skin” by engendering enduring molecular changes, such as in a person’s DNA methylation (DNAm). DNAm is sensitive to trauma exposure (for review, see Gladish et al., 2022) and is particularly plastic in early childhood (Pérez et al., 2019). Thus, measures of DNAm may serve as biomarkers of potential molecular plasticity in the context of interventions targeting the long-term effects of early-life adversity (Shonkoff et al., 2009).

One measure of DNAm is epigenetic age acceleration (EAA). DNAm at specific sites across the genome changes consistently and predictably with age. Thus, epigenetic age, or biological age, can be derived from DNAm at these age-associated sites. Discrepancies between someone’s biological and chronological age translate into “age acceleration,” with older biological age relative to chronological age indicating greater acceleration (Horvath, 2013). Although there are numerous epigenetic clocks, the pediatric-buccal-epigenetic (PedBE) clock is ideal for use in pediatric oral tissue samples because, unlike most other clocks, it was trained on pediatric data from noninvasive cheek swabs (L. M. McEwen et al., 2020; J. Wang & Zhou, 2021).

Greater exposure to early-life adversity is associated with greater EAA in pediatric samples (Copeland et al., 2022; Musci et al., 2023). Although this acceleration is potentially adaptive, because accelerated maturation may enhance fitness in stressful environments, acceleration may come at a physiological cost: For example, greater EAA is associated with prospective negative physical- and psychiatric-health outcomes in children (J. Wang & Zhou, 2021), including higher odds of a posttraumatic stress disorder diagnosis (Shenk et al., 2022), as well as greater anxiety and depression symptom severity (Zhang et al., 2023). Identifying interventions with the potential to prevent the onset of accelerated epigenetic aging is of great public-health importance. Such interventions may mitigate immediate and longer term disease risk, providing compelling social, ethical, and economical support for enhancing their accessibility.

Sensitive and responsive caregiving is among the most potent stress-buffering resources available to young children (Ainsworth, 1969; Bowlby, 1969; Hostinar et al., 2014). Children’s capacity to regulate stress emerges through dyadic processes, wherein caregivers coregulate, shape, and scaffold children’s coping skills (for review, see Hostinar et al., 2014). Thus, interventions that bolster caregiving offer a promising approach to attenuating adversity-induced changes in stress-sensitive biomarkers (for review, see Sullivan et al., 2024).

There is scarce research on parenting intervention effects on child epigenetic aging, and extant findings are mixed. Nine years after participating in a parenting and youth intervention the link between parental depressive symptoms and offspring EAA (measured at age 20) was attenuated among intervention youth relative to control youth (*n* = 399 Black youth living in rural Southern counties; Brody et al., 2016). However, in another study, 27 years after a nurse home-visiting infancy intervention or a control condition, EAA did not differ across treatment groups (*n* = 188 individuals born to low-income, young, or single mothers; O’Donnell et al., 2018). Finally, whereas a parenting intervention was not associated with group-level differences in preschool-aged children’s EAA, when data were collapsed across treatment and no-treatment groups, increases in positive parenting and decreases in negative parenting were associated with lower EAA 1.5 years later in children exposed to high levels of adversity (*n* = 62 predominantly Latino children with developmental delay; Sullivan et al., 2023). Importantly, the few available studies differ in populations, ages, duration between intervention and outcome assessment, and tissue type used to measure DNAm—all factors highly linked with DNAm (Jones et al., 2018) and, thus, EAA estimation—which complicates the interrelation of the mixed findings. Further, none of these studies collected baseline DNAm, precluding study of treatment-related EAA *changes*. Research using multiple time points of EAA is a critical next step.

Because early childhood is a sensitive period for the biological embedding of stress (Shonkoff et al., 2009), *early-childhood* parenting interventions may be highly impactful in improving prospective health (Clark et al., 2023; Heckman, 2006). Because children of color are systematically exposed to elevated stress (for review, see Iruka et al., 2022), it is also imperative to leverage interventions proven to be effective in diverse populations. Child–parent psychotherapy (CPP; Lieberman et al., 2015) is one such intervention, with robust evidence demonstrating effectiveness in enhancing the parent–child relationship of young children and their caregivers who have been exposed to trauma (for review, see Dozier & Bernard, 2023). Using a quasi-experimental repeated measures framework, the current study tested whether participation in the CPP intervention was associated with differences in pediatric biological aging using EAA assessed at baseline and follow-up. A low-income sample of mothers and their children engaged in CPP for child trauma exposure was compared to a low-income, weighted, propensity-matched community sample from the same metro region. We hypothesized that intervention participants would have less EAA at follow-up (10 months after intervention), consistent with slower biological aging compared with chronological age, relative to children in the comparison group.

**Statement of Relevance**

Experiencing stress early in life predicts future health problems. Biological aging and its acceleration relative to expectations for chronological age, measured in this study with accessible and noninvasive pediatric-buccal-epigenetic age acceleration, are biomarkers that may help scientists and health practitioners understand which people are at higher risk of poor future health. This study examined whether a psychosocial treatment designed to support safe, protective, and emotionally responsive parenting in caregivers and children who have experienced trauma—child–parent psychotherapy (CPP)—was associated with lower epigenetic age acceleration in early childhood compared with acceleration in a matched community group of children. Although epigenetic age acceleration was the same across groups before the intervention, after the intervention period, children who received CPP had lower epigenetic acceleration relative to the community-comparison group. The findings suggest that supporting healthy parent–child relationships may reduce children’s biological signs of stress exposure, potentially benefiting their future health.

## Open Practices Statement

No aspects of the study were preregistered. Study materials and analysis scripts are publicly available on GitHub at https://github.com/kobor-lab/Public-Scripts. The data are not publicly available because they include sensitive information related to epigenomics, trauma exposure, and therapy; requests for data can be sent to the corresponding authors.

## Method

### Participants

#### CPP group (intervention)

Biological mother-child dyads who were (a) referred for outpatient mental-health services because of the child’s exposure to interpersonal trauma (e.g., community violence, domestic violence, caregiver death) between 2013 and 2015 and (b) consented to receive CPP were invited to participate in a substudy to examine treatment-related changes in biological, behavioral, and psychological functioning. To be eligible, neither mother nor child could have a chronic medical condition (asthma, diabetes, heart/thyroid/growth problems, cancer, high blood pressure, autoimmune disease, or epilepsy) diagnosis or be taking daily medications used to treat asthma, heart or thyroid problems, or seizures; and mothers must not have reported active violence in the home or substance abuse of any kind. Of the 80 mothers approached about the substudy, 70 provided consent and enrolled. After the initial assessment, 21 dropped out of treatment, an additional three children refused the cheek-swab sample, and one child’s cheek swab failed quality control. The final clinical sample with two time points of buccal epithelial cell (BEC) epigenetic data included 45 families: 91.11% (*n* = 41) from the San Francisco site and 8.89% (*n* = 4) from Oakland (CPP: *n* = 45; age range = 3–6 years; 82.2% Latino). These mother and child dyads participated in up to 20 therapy sessions (*M* = 17.9, *SD* = 3.6); the mean duration between baseline and postintervention cheek-swab samples was 0.76 years (*SD* = 0.25); for demographic characteristics, see Table 1. The research team collected the second (posttreatment) buccal swab after 20 sessions or when participants ended treatment, whichever came first.

**Table 1.** Unweighted Sample Demographics by Group

Characteristic	CPP (*n* = 45)	Community (*n* = 110)
Children		
Age at first sample in years, *M* (*SD*)	4.28 (1.15)	4.09 (0.75)
Biological sex, % (*n*)		
Female	48.9 (22)	58.2 (64)
Male	51.1 (23)	41.8 (46)
Ethnicity, % (*n*)		
Latino^a^	82.2 (37)	40 (44)
Non-Latino	17.8 (8)	60 (66)
Adversity exposure,^b^ *M* (*SD*)	2.76 (1.60)	1.54 (1.58)
Mothers		
Age at enrollment in years, *M* (*SD*)	31.55 (6.34)	28.26 (5.93)
Race and ethnicity, % (*n*)		
Latina	82.2 (37)	38.2 (42)
Black	2.2 (1)	31.8 (35)
Asian American and Pacific Islander	2.2 (1)	2.7 (3)
White	6.7 (3)	9.1 (10)
Other or multiple races	6.7 (3)	18.2 (20)
Marital status at enrollment, % (*n*)		
Committed, married, or marriage-like	36.4 (16)	59.3 (64)
Separated, other, or multiple statuses	31.8 (14)	6.5 (7)
Single	31.8 (14)	34.3 (37)
Household annual income, % (*n*)		
< $5,000	9.1 (4)	15.5 (17)
$5,000–$11,999	40.9 (18)	6.4 (7)
$12,000–$14,999	6.8 (3)	9.1 (10)
$16,000–$24,999	15.9 (7)	20.9 (23)
$25,000–$34,999	13.6 (6)	6.4 (7)
$35,000–$49,999	4.5 (2)	13.6 (15)
$50,000-$74,999	—	13.6 (15)
$75,000–$99,999	—	5.5 (6)
$100,000–$149,999	2.3 (1)	7.3 (8)
$150,000–$199,999	4.5 (2)	0.9 (1)
≥ $200,000	2.3 (1)	0.9 (1)
Education, % (*n*)		
Less than a high school degree	42.2 (19)	4.6 (5)
High school degree or more	57.8 (26)	95.4 (103)

Note: Data are missing for age at enrollment (one CPP mother), marital status (one CPP mother and two community mothers), household annual income (one CPP mother), and education (two community mothers). CPP = child–parent psychotherapy. ^a^We use “Latino” to indicate the ethnicity of girls and boys; although gender-neutral terms such as “Latinx” and “Latine” are also appropriate, the participants in the current sample self-identified as Latino. ^b^Adversity exposure was assessed using the abbreviated Traumatic Events Screening Inventory at the first time point (T0).

CPP has demonstrated effectiveness in improving attachment quality, maternal behavioral-health outcomes, child psychological outcomes, and stress regulation (Dozier & Bernard, 2023; Hagan et al., 2021; Lieberman et al., 2005), making it an ideal intervention for testing the potential resilience-promoting impact of enhanced parent–child relationship quality on children’s EAA. In CPP, clinicians work with caregivers to create physical and emotional safety after traumatic events, which enhances attachment quality, reduces child trauma symptoms, and promotes child regulation (Dozier & Bernard, 2023; Lieberman et al., 2005). During weekly 1-hr sessions, clinicians guide dyads through developmentally appropriate social interactions (including free play), supporting caregivers in understanding how their child’s behavior and traumatic experiences are related and in helping their child process those experiences. Clinicians use strategies such as playing with toys related to the traumatic experience, insight-oriented reflections, and unstructured and reflective developmental guidance (for further details on the intervention, see Lieberman et al., 2005, 2015; Lieberman & Van Horn, 2005).

#### Stress, Eating, and Early Development study group (comparison)

The community-comparison sample was drawn from the same urban region from a cohort of biological mother-child dyads enrolled in the longitudinal Stress, Eating, and Early Development (SEED) study, which was designed to assess the effects of prenatal factors, including high levels of stress, on children’s development (for details, see Bush et al., 2017; see also Table 1). Eligible women were between 18 and 45 years of age, between 8 and 23 weeks pregnant with a singleton, had a body mass index between 25 and 40 kg/m^2^, reported incomes less than 500% of the federal poverty level (75% reported ≤ 200%), and had live births. Children with buccal-swab data available from at least two of the available time points (age 3, 4, and 5 visits) were used for this analysis to optimize matching child ages in the CPP study. Of the 162 women enrolled in the SEED study, 148 families completed at least one of those assessments; 138 of these families (93.24%) provided at least one time point of a child cheek-swab sample. However, because the current study required children to have at least two time points of buccal data to parallel the pre-post design of the treatment group, the final matched comparison-group analytic sample totaled 110 SEED study participants.

### Measures

Childhood adversity was measured in both cohorts at the first time point using the Traumatic Events Screen Inventory (TESI; Ford et al., 2002) in the CPP intervention group and an abbreviated screener in the comparison group that omitted reports of child sexual or physical abuse (because of mandatory reporting requirements and the lack of clinical services offered in the community-sample study). Harmonized scoring of the abbreviated item list, assessing exposure to 13 traumatic event types, was used in the analyses.

### Procedures

All participants provided written consent. The institutional review boards of the University of California, San Francisco; San Francisco General Hospital; and Benioff Children’s Hospital Oakland approved all study procedures. Two trained bicultural, bilingual research assistants collected anthropometric and biological data at the hospital clinics.

#### Biological sample collection and preparation

Cheek swabs were collected, sealed, and shipped to the University of British Columbia for DNA extraction and DNAm microarray profiling with the Illumina EPIC v1.0 array. All samples were randomized together on cohort (CPP or SEED), sex, age at sample collection, TESI score, Latino identity, and time point (T0/T1) to reduce the likelihood of confounding with technical batch effects. Samples were prepared, extracted, and bisulfite-converted using previously established methods (Merrill et al., 2021).

#### DNAm microarray preprocessing

Background subtraction and color correction were performed with *noob* in the *minfi* R package for all buccal samples (Fortin et al., 2016). In addition, sample quality was confirmed using metrics in the *minfi* and *ewastools* R packages (Aryee et al., 2014; Murat et al., 2020). Ultimately, 110 children from the SEED group and 45 children from the CPP group had at least two time points of buccal samples. Because some SEED participants could have contributed three time points of buccal samples, the total number of samples available for analysis was 424. Using these samples, estimated BEC proportion—the primary cell type in cheek swabs—was inferred using the *EpiDISH* R package (Zheng et al., 2019), because cell type is a major driver of DNAm (Jones et al., 2018). For additional data processing and cell type proportion estimation, see the Supplemental Material available online.

#### EAA estimation

Epigenetic age was calculated using the PedBE clock (L. M. McEwen et al., 2020; https://github.com/kobor-lab/Public-Scripts/blob/master/PedBE.Md), which was highly correlated with chronological age, *r* = .89, 95% confidence interval (CI) = [.85, .92], *t*(308) = 24.70, *p* < 2.2 × 10^−16^. PedBE age was then regressed onto chronological age, controlling for estimated BEC proportions, and residuals from this model constituted PedBE age acceleration.

We also calculated EAA as a sensitivity analysis using the Horvath PanTissue clock. Although this clock developed in adults is less appropriate than the PedBE clock for these analyses, it is a highly cited and used tool in the field (Binder & Horvath, 2022), allowing for the comparison of previous findings. Further, none of the DNAm sites used in estimating the Horvath pan-tissue clock overlap with the PedBE clock, providing a robust comparator. EAA from the Horvath PanTissue clock (Horvath, 2013) was calculated using the *methylclock* package (Pelegí-Sisó et al., 2021). This clock was less accurate than the PedBE clock but still correlated with chronological age, *r* = .64, 95% CI = [.57, .70], *t*(308) = 14.77, *p* < 2.2 × 10^−16^. As above, EAA was calculated as the residuals from a regression of estimated epigenetic age on chronological age.

### Propensity matching and weighting

Randomizing trauma-exposed children to receive the well-established, efficacious CPP treatment raises ethical concerns. Quasi-experimental methods offer a methodologically robust alternative to testing causal inferences in such situations (Baldwin et al., 2023). In this study, we used propensity-score methods to identify and weight a community-comparison group (SEED) well matched to the intervention group (CPP). We used the *OptMatch* package (Gong et al., 2020) using full matching propensity weighting with caliper restriction using logistic regression (Austin & Stuart, 2017) to match community-comparison children to the treatment-group children. Children were matched on key variables considered integral to either longitudinal DNAm changes or the population comparison across treatment and matched community-comparison groups: age at first biological sample collection, time between biological sample collections, sex, difference in BEC proportion between biological samples, child adversity score from the abbreviated TESI at T0, and child Latino identity. We elected to leverage full matching methods given our quasi-experimental approach. This approach also eliminates the bias of *k*:1 matching, which occurs when a specific number of children are forced to match with each individual in the treatment group, even if some children may lack a perfect match in the comparison cohort (Austin & Stuart, 2017; Rosenbaum & Rubin, 1985). Additionally, because there was more than one comparison child matched for each treatment child, we used the recommended optimal caliper restriction of 0.2 (Austin, 2011; Y. Wang et al., 2013) to improve the quality of inferences. These restrictions mandate matching only children who are highly similar on all criteria. Together, this procedure weights each child in the matching group so the matching group, as a whole, is as similar as possible to the treatment group on the matching variables.

First, we selected participants with two biological samples. Because there were several children in the SEED group with three samples collected, a preliminary round of full matching that included multiple DNAm sample pairs from the same child was performed on all SEED individuals with at least two time points to match to the treatment-group individuals. This was done because each T0 to T1 comparison best matched to the treatment group would be prioritized, and no individual would be present in the analysis more than once. All SEED individuals who were matched multiple times were selected to be removed on the basis of the lowest weighted combination or, if weighted the same, randomly selected. This resulted in a total of two time points (T0 and T1) of DNAm data for 45 treatment-group children and 110 matched community-comparison children, totaling 155 unique children for analysis.

The community-comparison children were matched to the children who received CPP on the above metrics using full matching with caliper restriction to create a weighted data set, with these weights being used in all subsequent statistical analyses (Austin & Stuart, 2017). All variables were well matched between the two data sets with this method, except for Latino identity, which was still more likely in the treatment group than the community-comparison group, χ^2^(2, 155) = 6.90, *p* = .008, Cramér’s *V* = .21, although less so than before the matching, χ^2^(2, 155) = 43.96, *p* = 3.34 × 10^−11^, Cramér’s *V* = .53. For a visual depicting absolute standardized mean differences for child demographic characteristics before and after matching, see Figure S1 in the Supplemental Material. As depicted in Figure S1, matching procedures resulted in children being well balanced across groups on the selected matching variables, making these groups appropriate for comparison.

### Statistical analysis

To estimate the potential association between treatment and PedBE age acceleration, we evaluated group differences using two weighted analyses of covariance (ANCOVAs): one comparing differences in BEC proportion-adjusted PedBE age acceleration at T0 and one at T1. ANCOVAs used propensity-matching weights and were adjusted for all covariates used in the matching process (i.e., age at T0, sex, Latino identity, duration of time between biological sample collections, and T0 child adversity exposure) as recommended (Austin & Stuart, 2017).

Additionally, a sensitivity analysis repeated this procedure for the estimated Horvath PanTissue EAA at each time point. Finally, to determine whether any differences were potentially sensitive to the remaining imbalance of Latino-identifying children between the otherwise well-matched treatment and community groups, models with an interaction of group (treatment or community comparison) and Latino identity (yes/no) were examined for both PedBE age acceleration and Horvath PanTissue EAA at T0 and T1.

## Results

### Demographic and covariate descriptives

Table 1 presents unweighted mother and child demographic characteristics separated by treatment group. Before propensity matching and weighting procedures, children were similar in age, sex distribution, and adversity exposure. Caregivers reported a wide range of adversity exposure subtypes for their children, and frequencies were not statistically different across treatment and comparison groups in nearly all categories (see Table S1 in the Supplemental Material), providing further evidence of similarities across the two groups. A larger proportion of mothers in the intervention group identified as Latina, endorsed separated/other marital status, and had lower annual incomes and educational attainment relative to mothers in the community-comparison group. Figure S1 displays absolute standardized mean differences for child demographic characteristics before and after propensity-score weighting.

Figure S2 displays bivariate correlations between the abbreviated TESI score and baseline and posttreatment EAA calculated with both PedBE and Horvath PanTissue clocks. In this sample of 3- to 6-year-old children exposed to high adversity, trauma exposure was unrelated to either estimate of EAA at either time point. Collapsing across treatment groups, correlations indicated low levels of associations among covariates of interest (see Fig. S3). Notably, older children and children endorsing Latino identities had higher adversity scores and shorter time durations between cheek-swab collection points. A longer duration between swab collections was associated with a greater change in predicted BEC proportion (inferred from cheek-swab DNAm), as expected (Wong et al., 2022).

### Postintervention, children participating in CPP had less EAA using both PedBE and Horvath PanTissue clocks than the matched community-comparison group

**Fig. 1.**
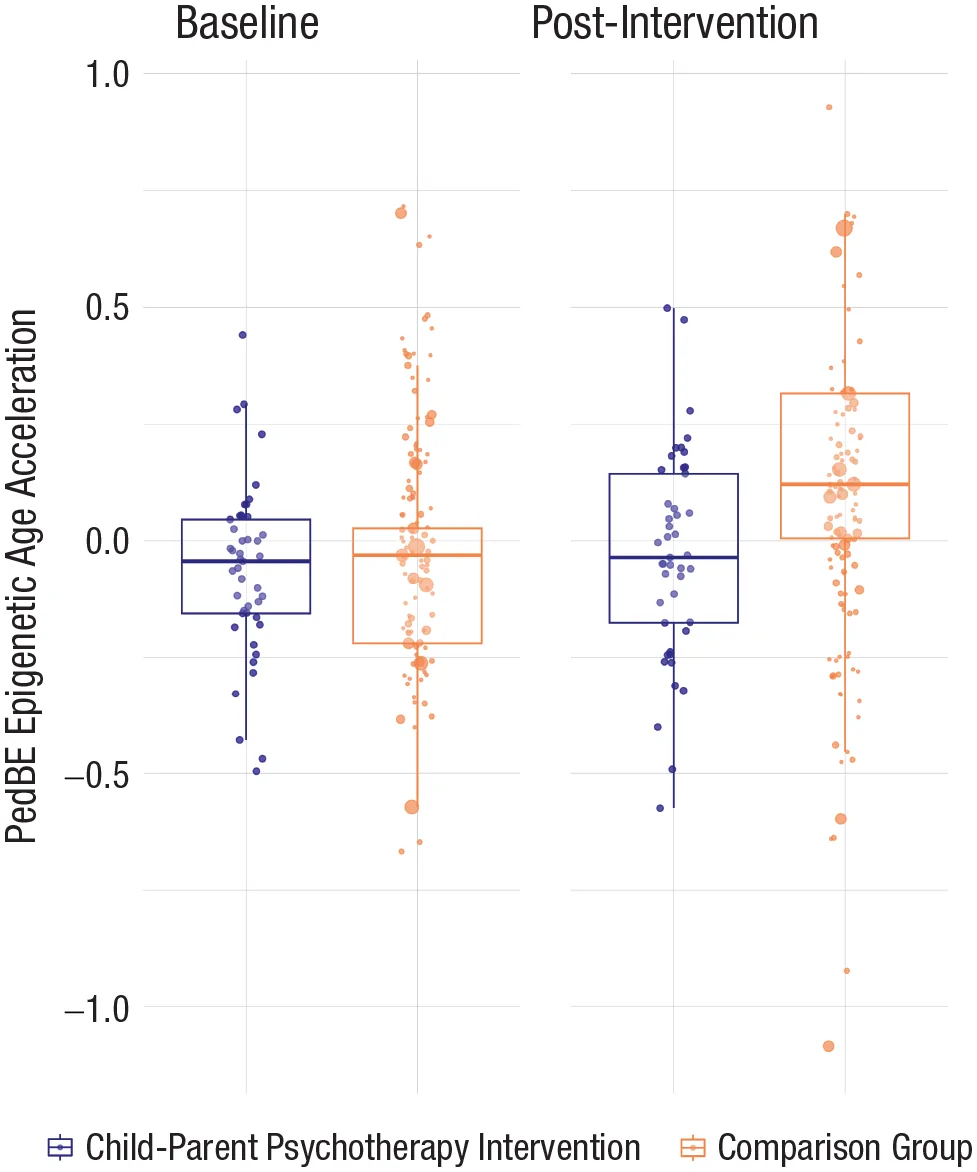
Box plot of PedBE age acceleration at baseline and postintervention time points for children who received CPP and those who did not. Propensity-weighted PedBE age acceleration (corrected for BEC proportion) was equal between both the treatment and matched community-comparison groups at baseline. However, there was a significant, although small, effect size difference between the treatment and community-comparison condition postintervention. Postintervention, PedBE age acceleration was greater for those children from the matched community-comparison sample relative to those who received intervention. The point size for each individual participant (dots on figure) reflects propensity-score weighting in the comparison group. In the CPP group, all samples were equally weighted. PedBE = pediatric-buccal-epigenetic; CPP = child–parent psychotherapy; BEC = buccal epithelial cell.

Figure 1 illustrates the findings. At T0, after adjusting for covariates (age at T0, sex, Latino identity, duration between biological sample collection, and T0 child adversity exposure), BEC proportion-adjusted PedBE age acceleration was the same across CPP treatment (μ = −0.06) and propensity-matched and weighted community (μ = −0.06) samples, *F*(1, 148) = 0.03, *p* = .854, Cohen’s *f* = 0.0, 95% CI = [0.00, 1.00]. However, the two groups differed at T1: After the intervention (approximately 10 months after baseline), children in the treatment group (μ = −0.03) had less PedBE age acceleration (slower rate of biological aging) than children in the matched community-comparison group (μ = 0.11), *F*(1, 148) = 6.39, *p* = .013, Cohen’s *f* = 0.21, 95% CI = [4.90 × 10^−3^, 1.00].

In the sensitivity analysis to consider whether findings were clock-specific, substituting EAA calculated with the Horvath PanTissue clock as the outcome, findings paralleled PedBE analyses: At T0, EAA was not significantly different between the treatment (μ = −0.04) and matched community (μ = 0.05) group, *F*(1, 148) = 1.13, *p* = .290, Cohen’s *f* = 0.087, 95% CI = [0.00, 0.05] (see Fig. S4). At T1, however, the groups differed; Horvath PanTissue EAA was lower in the treatment group (μ = −0.18) relative to the community group (μ = 0.07), *F*(1, 148) = 7.70, *p* = .006, Cohen’s *f* = 0.23, 95% CI = [0.01, 0.13]. Although the groups were not balanced for Latino identity, there was no interaction between Latino identity and treatment on EAA.

## Discussion

Although it is well established that evidence-based dyadic interventions can reduce the psychological sequelae of early trauma exposure (Toth et al., 2015), it remains unclear whether dyadic interventions support child health assessed at the molecular level (Sullivan et al., 2024). Findings from this study indicate that dyadic intervention is associated with lower levels of EAA posttreatment, with results replicating across two different molecular assessments of accelerated aging (PedBE and Horvath PanTissue). Findings provide robust, quasi-experimental support that dyadic intervention is associated with trauma-related accelerated-aging biomarkers, most likely in a direction beneficial for health and development.

Findings suggest that CPP supported slower biological aging, likely through promoting safe, protective, and emotionally responsive caregiving (e.g., Sullivan et al., 2023). These quasi-experimental findings allow inference regarding *how* caregiving can influence linkages between adversity exposure and child health. Whereas early adversity leads to and accelerates disease processes (Grummitt et al., 2021), supporting caregivers in enhancing their parenting appears to have the potential to alter developmental biology sensitive to stress (for review, see Thumfart et al., 2022). Among CPP’s primary intervention targets is improving attachment quality, and CPP enables caregivers to better help their children regulate when experiencing stress (including posttraumatic stress; Lieberman et al., 2015). Aligning with models of parental buffering of stress exposure (e.g., Hostinar et al., 2014; Sullivan et al., 2024), this slower biological age acceleration may reflect caregivers’ enhanced capacity to meet their child’s needs for safety and security (Roubinov et al., 2021) and, in turn, may support future positive health. Given links between child mental-health symptoms and EAA (Zhang et al., 2023), improved caregiver mental health is another potential mechanism of intervention effects found here. CPP also reduces caregiver psychiatric symptoms (Lieberman et al., 2005), which, in turn, have been associated with improvements in child mental health posttreatment (Hagan et al., 2017) and thus may be among the constellation of factors that resulted in slower child biological aging.

The replication of results across the PedBE and Horvath PanTissue clocks increases our confidence in study findings because these tools share no overlapping DNAm sites (Horvath, 2013; L. M. McEwen et al., 2020). However, these data also illustrate the importance of using pediatric-specific biomarkers for studies of early-life interventions. The PedBE clock was developed exclusively in pediatric populations using specimens collected with cheek swabs. This noninvasive method (relative to blood) is ideal when working with protected populations (L. M. McEwen et al., 2020). In the current study, PedBE-predicted age was more closely associated with children’s chronological age (*r* = .89) than Horvath PanTissue-predicted ages (*r* = .64). Further, PedBE-predicted ages have a median error of approximately 4 months relative to the median error of approximately 3 years in Horvath PanTissue-predicted ages calculated in pediatric samples. Importantly, the PedBE error is smaller than the approximately 10-month span between biological samples (over which CPP occurred), supporting the validity of these observed effects (L. M. McEwen et al., 2020). Although the replication of findings across clocks was a useful sensitivity analysis, the greater accuracy of the PedBE clock in our study highlights the importance of using tools specifically designed for pediatric samples when evaluating intervention effects on children.

Bivariate correlations indicated that EAA was not associated with an abbreviated TESI score at either time point. Although adversity exposure is often found to be associated with cellular aging in adults and in samples with broad variability in stress exposure (such as the general population; Gladish et al., 2022), the lack of association found here is not surprising because the high level of adversity exposure present in the two low-income samples may engender a potential ceiling effect. The null correlation may also result from the narrow age band of the young children studied here relative to other studies that have examined broader age bands (e.g., 6–13 years; Jovanovic et al., 2017), resulting in greater age-related variability in both time to have experienced adversity and in cellular aging.

The findings reported here provide a foundation for future research into intervention effects on pediatric biological aging. Although a randomized controlled trial would provide the most compelling evidence of causality, the quasi-experimental design used in this study supports considerations of causality in the association between adversity and altered developmental biology sensitive to stress (Baldwin et al., 2023). Whereas this effect may be small in magnitude (Cohen’s *f* = 0.21), the possible biological significance of lower EAA in early childhood is considerable given developmental trajectories of health and disease (Rutter et al., 2006). Indeed, early intervention may have the greatest impact on child and later-life outcomes (Clark et al., 2023; Heckman, 2006). Study findings suggest family-based psychosocial interventions for children exposed to trauma have the potential to slow rates of biological aging. Slower biological aging may subsequently promote differences in prospective health trajectories.

### Strengths and limitations

Regarding strengths, this study is the first known intervention trial to present pre- and post-EAA data, using two epigenetic clocks, on children who have participated in dyadic psychotherapy—existing research either uses long-term follow-up data from young adults or examines only posttreatment biological data. Importantly, participants received variable dosages of treatment, with some receiving < 20 sessions (e.g., because of lifestyle factors or moving residences). Thus, results reflect a therapeutic experience aligned with real-world treatment. Such effectiveness research is a critical component of widely translating intervention research into practice (Glasgow & Estabrooks, 2018). Further, low-income families, with a strong representation of Latino families, comprised the study population, providing evidence from a critically understudied, growing sector of the U.S. population that is frequently exposed to elevated levels of stress (Heard-Garriss et al., 2021). Using propensity-score matching contributes robust, quasi-experimental evidence for a population of young children for whom the potential ramifications of randomization to a control treatment is ethically tenuous. Taken together, it is tempting to speculate about the potential causality of early, dyadic intervention to slow EAA of young trauma-exposed children. Because EAA predicts poor health outcomes in adults, with emerging evidence in children (Musci et al., 2023), policymakers and practitioners alike may find that these biological results provide compelling support for increasing families’ access to dyadic psychotherapy to promote improved prospective health.

Regarding limitations, we acknowledge these quasi-experimental analyses are not equivalent to a randomized control study, which is typically unethical in the context of treating families seeking therapy for trauma exposure. Further, although our study had a considerable sample size for an intensive young-child trauma-therapy study, and our matching procedures enabled utilization of a full sample of *N* = 155, the intervention-group sample size was modest (*n* = 45). Accordingly, inferences must be considered cautiously, and replication in a larger sample would increase confidence in the findings. Moreover, although we draw from literature on CPP and developmental psychopathology to identify likely mechanisms of action facilitating lower EAA (e.g., enhanced parent–child relationship), we did not assess changes in parenting or relationships in this study. Future research should incorporate robust measurements of parenting and attachment quality to test this potential mechanism. We also acknowledge that, although the full propensity matching was successful on most demographic features, the two groups did not match on Latino identity. Further, the Horvath PanTissue clock underestimated epigenetic age in Latino children, consistent with previous studies (Horvath et al., 2016); however, caregiver-reported ethnicity was adjusted in all analyses and did not moderate treatment effects. Future studies should examine whether ethnic identity and cultural context or the differences in underlying genetic architecture associated with genetic ancestry could contribute to these findings (Chan et al., 2023). These findings may be tissue-specific, and future studies should endeavor to examine whether similar EAA trends are present across tissues. Finally, although findings suggest intervention may be associated with less EAA, further longitudinal work is needed to determine whether these differences are related to prospective physical- and behavioral-health outcomes.

### Conclusion

Findings intersect historically siloed disciplines of biology and psychology, suggesting psychosocial therapy may attenuate the biological embedding of stress in young children exposed to trauma. Further, findings indicate that early-life adversity exposure is not deterministic of poor biological health, relaying a hopeful message for stress-exposed families and for the providers and systems who endeavor to support their well-being. Thus, dyadic interventions may foster child resilience and promote psychiatric and physical health over time.

## Supplemental Material

sj-docx-1-pss-10.1177_09567976241260247 – Supplemental material for Intervening After Trauma: Child–Parent Psychotherapy Treatment Is Associated With Lower Pediatric Epigenetic Age Acceleration Supplemental material, sj-docx-1-pss-10.1177_09567976241260247 for Intervening After Trauma: Child–Parent Psychotherapy Treatment Is Associated With Lower Pediatric Epigenetic Age Acceleration by Alexandra D. W. Sullivan, Sarah M. Merrill, Chaini Konwar, Michael Coccia, Luisa Rivera, Julia L. MacIsaac, Alicia F. Lieberman, Michael S. Kobor and Nicole R. Bush in Psychological Science

## Transparency

*Action Editor*: Daniela Schiller

*Editor*: Patricia J. Bauer

*Author Contributions*

**Alexandra D. W. Sullivan and Sarah M. Merrill** contributed equally to the writing of this manuscript, and they share first authorship.

**Alexandra D. W. Sullivan:** Investigation; Writing – original draft; Writing – review & editing.

**Sarah M. Merrill:** Conceptualization; Data curation; Formal analysis; Methodology; Validation; Visualization; Writing – original draft; Writing – review & editing.

**Chaini Konwar:** Data curation; Formal analysis; Methodology; Writing – review & editing.

**Michael Coccia:** Data curation; Formal analysis; Methodology; Validation; Visualization; Writing – review & editing.

**Luisa Rivera:** Data curation; Investigation; Writing – review & editing.

**Julia L. MacIsaac:** Data curation; Investigation; Writing – review & editing.

**Alicia F. Lieberman:** Conceptualization; Funding acquisition; Investigation; Methodology; Project administration; Supervision; Writing – review & editing.

**Michael S. Kobor:** Conceptualization; Data curation; Funding acquisition; Methodology; Project administration; Resources; Supervision; Writing – review & editing.

**Nicole R. Bush:** Conceptualization; Data curation; Funding acquisition; Investigation; Methodology; Project administration; Resources; Supervision; Writing – original draft; Writing – review & editing.
