# Supplementary material for: Intervening After Trauma: Child–Parent Psychotherapy Treatment Is Associated With Lower Pediatric Epigenetic Age Acceleration
Source: Psychol Sci. 2024 Aug 14;35(9):1062–73. doi: 10.1177/09567976241260247 (PMC13614526; doi:10.1177/09567976241260247)
Supplement: Supplementary file 1 [file sj-docx-1-pss-10.1177_09567976241260247.docx]

# Supplemental Materials

## *Methods*

### 1. *DNA Methylation Outlier Sample Removal*

In addition to *minfi* and *ewastools* package control metrics for sample outlier removal, sex mismatch after sex was estimated using the *getSex* function from the *minfi* package. Also, samples with >2% poorly performing samples based on detection *p*-value using the *detectionP* function from the *minfi* package, and outliers identified the *outlyx* function from the *wateRmelon* package were removed from the sample as outliers before epigenetic age calculation or cell type estimation. In total, 23 samples were removed as poorly performing outliers based on these metrics. As some maternal samples were also included in randomization, samples were subset to children’s samples only.

### 2. *DNA Methylation Cell Type Proportion Estimation*

A primary factor in individual DNA methylation variability is differential cell type proportions (Jones et al., 2018). While cheek swabs primarily consist of buccal epithelial cells (BEC) in pediatric populations (Theda et al., 2018), these are still heterogenous samples. Therefore, the *hepidish* function, using robust partial correlation, in the *EpiDISH* package was used to estimated cell type proportions (Zheng et al., 2019). All included samples had an estimated BEC proportion greater than 49%, a maximum of 99%, and a mean proportion of 88%. There was no association between group and estimated BEC proportion (F(1, 423) = 2.36, p = 0.125).

## Results

### 1. *No interaction with Latino status in PedBE findings. While Horvath pantissue consistently predicted Latino children to be younger, this did not account for the main effect of intervention*

There were no significant interactions between treatment participation and endorsement of Latino identity for any timepoint using PedBE EAA, nor at T0 using Horvath pantissue EAA. However, there was a significant interaction between treatment participation and Latino identity at T1 (F(1, 148) = 7.35, p = 0.007; Cohen’s f = 0.22, 95% CI [0.01, 0.11]), with children endorsing Latino identities having less Horvath pantissue EAA than those who did not, regardless of intervention group. However, even accounting for this interaction, there is still a main effect of intervention participation (F(1, 148) = 8.07, p = 0.005; Cohen’s f = 0.23, 95% CI [0.00, 0.04]).

*Supplemental Figures*

Supplemental Figure 1. A Love plot of absolute standardized mean differences before and after propensity score matched weighting on model variables. Points within the two solid vertical lines at the left of the graph represent variables with absolute standardized mean differences between 0 and 0.10. This implies an acceptable balance between the groups. To the left of the dashed vertical line (<0.05 absolute standardized mean differences) implies a good match between the groups.

Supplemental Figure 2. Parent-reported abbreviated traumatic events screening inventory (TESI) score at baseline did not associate at either timepoint with epigenetic age acceleration (EAA) measured with the (A) PedBE clock or (B) Horvath pantissue clock. Individuals are colored by their propensity score weight in the model, and there is visible pattern in propensity score weight distribution.

Supplemental Figure 3. A heatmap of Pearson correlations among study covariates. Darker colors indicated a stronger correlation between variables. All covariates in the models were correlated 0.2 or less.

Supplemental Figure 4. Boxplot of pediatric epigenetic age acceleration (Horvath PanTissue) (corrected for BEC proportion) at baseline and post-intervention timepoints for those who received child-parent psychotherapy (CPP) and those who did not. Point size for each individual participant (dots on figure) reflects propensity score weight in comparison group. In the CPP group, all samples were equally weighted.

| Abbreviated Traumatic Events Screening Inventory Item Content | CPP (n=45)  % endorsed | Community (*n*=110)  % endorsed |
| --- | --- | --- |
| Involved in a serious accident | 4.44% | 11.11% |
| Observed a serious accident | 11.11% | 3.97% |
| Been in a natural disaster | 2.22% | 1.59% |
| Experienced the illness or injury of someone close | 26.67% | 18.25% |
| Experienced the death of someone close | 22.73% | 11.11% |
| Received a serious medical procedure or treated in the emergency room | 22.22% | 33.33% |
| Experienced separation from a caregiver under stressful circumstances* | 64.44% | 38.10% |
| Was present when a family member was mugged | 11.11% | 3.20% |
| Experienced an animal attack | 20.00% | 8.73% |
| Known or seen that a family member was incarcerated* | 40.00% | 11.90% |
| Seen or heard others' violence | 34.09% | 22.22% |
| Exposed to war or terrorism | 0.00% | 1.59% |
| Seen or heard acts of war or terrorism | 20.00% | 25.40% |
| Note: * = proportions are statistically different at *p* < .001 using the 2-sample test for equality of proportions | | |

*Supplemental Table.*

Supplemental Table 1.

*3. Supplemental References*

For further detail on propensity score matching methods, refer to Austin, 2014.

For further detail on CPP, refer to Cicchetti et al., 2006; Lieberman et al., 1991, 2005; Toth et al., 2002.

For further detail on relevant data processing techniques, refer to England-Mason et al., 2022; Merrill et al., 2021; Moore et al., 2021.

Austin, P. C. (2014). A comparison of 12 algorithms for matching on the propensity score. *Statistics in Medicine*, *33*(6), 1057–1069. https://doi.org/10.1002/sim.6004

Cicchetti, D., Rogosch, F. A., & Toth, S. L. (2006). Fostering secure attachment in infants in maltreating families through preventive interventions. *Development and Psychopathology*, *18*(3), 623–649. https://doi.org/10.1017/S0954579406060329

England-Mason, G., Merrill, S. M., Gladish, N., Moore, S. R., Giesbrecht, G. F., Letourneau, N., MacIsaac, J. L., MacDonald, A. M., Kinniburgh, D. W., Ponsonby, A. L., Saffery, R., Martin, J. W., Kobor, M. S., & Dewey, D. (2022). Prenatal exposure to phthalates and peripheral blood and buccal epithelial DNA methylation in infants: An epigenome-wide association study. *Environment International*, *163*, 107183. https://doi.org/10.1016/J.ENVINT.2022.107183

Jones, M. J., Moore, S. R., & Kobor, M. S. (2018). Annual Review of Psychology Principles and Challenges of Applying Epigenetic Epidemiology to Psychology. *Annual Review of Psychology*, *69*, 459–485. https://doi.org/10.1146/annurev-psych-122414

Lieberman, A. F., Van Horn, P., & Ippen, C. G. (2005). Toward evidence-based treatment: Child-parent psychotherapy with preschoolers exposed to marital violence. *Journal of the American Academy of Child and Adolescent Psychiatry*, *44*(12), 1241–1248. https://doi.org/10.1097/01.chi.0000181047.59702.58

Lieberman, A. F., Weston, D. R., & Pawl, J. H. (1991). Preventive intervention and outcome with anxiously attached dyads. *Child Development*, *62*(1), 199–209. https://doi.org/10.1111/j.1467-8624.1991.tb01525.x

Merrill, S. M., Moore, S. R., Gladish, N., Giesbrecht, G. F., Dewey, D., Konwar, C., Macissac, J. L., Kobor, M. S., & Letourneau, N. L. (2021). Paternal adverse childhood experiences: Associations with infant DNA methylation. *Developmental Psychobiology*, *63*, 22174. https://doi.org/10.1002/dev.22174

Moore, S. R., Merrill, S. M., Sekhon, B., Macisaac, J. L., Kobor, M. S., Giesbrecht, G. F., Letourneau, N., & Team, A. (2021). *Infant DNA methylation: An early indicator of intergenerational trauma?* https://doi.org/10.1016/j.earlhumdev.2021.105519

Theda, C., Hwang, S. H., Czajko, A., Loke, Y. J., Leong, P., & Craig, J. M. (2018). Quantitation of the cellular content of saliva and buccal swab samples. *Scientific Reports 2018 8:1*, *8*(1), 1–8. https://doi.org/10.1038/s41598-018-25311-0

Toth, S. L., Maughan, A., Manly, J. T., Spagnola, M., & Cicchetti, D. (2002). The relative efficacy of two interventions in altering maltreated preschool children’s representational models: Implications for attachment theory. *Development and Psychopathology*, *14*(4), 877–908. https://doi.org/10.1017/s095457940200411x

Zheng, S. C., Breeze, C. E., Beck, S., Dong, D., Zhu, T., Ma, L., Ye, W., Zhang, G., & Teschendorff, A. E. (2019). EpiDISH web server: Epigenetic Dissection of Intra-Sample-Heterogeneity with online GUI. *Bioinformatics*, *36*(6), 1950–1951. https://doi.org/10.1093/bioinformatics/btz833
